# Exploring Knowledge About Dialysis, Transplantation, and Living Donation Among Patients and Their Living Kidney Donors

**DOI:** 10.1007/s12529-015-9461-7

**Published:** 2015-01-30

**Authors:** Lotte Timmerman, Sohal Y. Ismail, Annemarie E. Luchtenburg, Willij C. Zuidema, Jan N. M. IJzermans, Jan J. V. Busschbach, Willem Weimar, Emma K. Massey

**Affiliations:** 1 Department of Internal Medicine, Section Nephrology & Transplantation, Erasmus Medical Center Rotterdam, P.O. Box 2040, 3000 CA Rotterdam, The Netherlands; 2 Psychiatry, Section Medical Psychology and Psychotherapy, Erasmus Medical Center Rotterdam, P.O. Box 2040, 3000 CA Rotterdam, The Netherlands; 3 General Surgery, Erasmus Medical Center Rotterdam, P.O. Box 2040, 3000 CA Rotterdam, The Netherlands

**Keywords:** Informed consent, Kidney transplantation, Knowledge, Living donor, Organ donation, Questionnaire

## Abstract

**Background:**

In order to make a well-considered decision and give informed consent about renal replacement therapy, potential living kidney donors and recipients should have sufficient understanding of the options and risks.

**Purpose:**

We aimed to explore knowledge about Dialysis & Transplantation (DT) and Living Donation (LD) among prospective living kidney donors and recipients.

**Methods:**

Eighty-five donors and 81 recipients completed the Rotterdam Renal Replacement Knowledge-Test (R3K-T) 1 day before surgery. The questionnaire was available in various languages.

**Results:**

Recipients knew significantly more about DT than donors (*p* < 0.001); donors knew more about LD than recipients (*p* < 0.001). A minority of donors (15 %) and recipients (17 %) had a score that was comparable to the knowledge level of the naïve general population. Recipients and donors knew less about DT and LD if their native language was not Dutch. In addition, recipients knew less about DT if they were undergoing pre-emptive transplantation.

**Conclusions:**

We conclude that recipients and donors retain different information. The decision to undergo living donation appears to be not always based on full knowledge of the risks. We recommend that professionals assess knowledge of prospective donors and recipients during the education process using the R3K-T, and extra attention is required for non-native speakers.

## Introduction

Live donor kidney transplantation is the best option for extending and improving the lives of patients with end-stage renal disease (ESRD) [1, 2]. For the living donor, the short-term medical outcomes are well documented: the overall mortality rate is 0.03 % [3, 4] and the morbidity rate (including minor complications) is <10 % [5]. Two recent studies showed an increased risk of ESRD among living kidney donors on the long term after donation [6, 7]; however, the majority of studies revealed that donors have a normal life span and an excellent quality of life many years after donation [8–11].

Although some have argued that the living donation procedure goes against the medical ethical principle of primum non nocere or “first do no harm” [12], it is justified for two reasons. Firstly, the benefits for the donor are a justification, such as an increased quality of life [13]. Secondly, the right to autonomy [14], which comprises that individuals have the right to determine what they do and what happens to their body.

In order to make a well-considered autonomous decision regarding living kidney donation, it is important that the potential donor is fully informed about the consequences of the donation/transplantation procedure and other renal replacement therapies (RRT) and the decision is consistent with the person’s values [15]. In addition, the potential donor should be willing to donate, medically and psychosocially suitable, and free from coercion [16]. These components constitute the “informed consent” [16]. For every medical treatment, informed consent is important in order to guarantee a patient’s autonomy [17]. Informed consent is particularly important among living donors since the right to autonomy is one of the justifications of this procedure as mentioned above [14].

However, two studies have shown that some living kidney donors do not completely consider the risks versus the benefits of the donation [18, 19]. Moreover, a retrospective study by Valapour and colleagues [20] revealed that a substantial percentage of donors reported after donation that they had not completely understood the psychological, financial, and long-term medical risks of donation at the time of their surgery. What is yet unknown is the actual level of knowledge on dialysis, transplantation, and living donation among prospective living kidney donors at the time of the donation and the factors that are associated with their knowledge. This question is relevant to examine whether professionals should make extra efforts (in particular cases) to ensure that the prospective donor makes a well-informed decision.

Like potential donors, patients with ESRD need appropriate knowledge about dialysis, transplantation, and living donation to make a fully informed treatment decision [21]. Therefore, we also investigated the knowledge level of prospective recipients. This examination is also relevant as a lack of knowledge among ESRD patients is probably related to concerns regarding living donation [22] and a barrier to pursuing live donor kidney transplantation [23–25]. In reaction to this, various educational interventions have been developed to increase knowledge and indirectly to promote live donor kidney transplantations [23]. Insight into the current gaps in recipients’ knowledge and the factors that are associated with knowledge are relevant for improving such interventions.

In the present study, we explored the level of knowledge about dialysis, transplantation, and living donation among prospective living kidney donors and recipients using the Rotterdam Renal Replacement Knowledge-Test (R3K-T): [26–28] at a moment in time when they should be fully informed yet still be unbiased by the experience. Our aim was to examine whether there were gaps in knowledge among prospective donors and recipients. We also aimed to examine whether knowledge differed between donors and recipients and the sociodemographic and medical factors that are associated with knowledge.

## Materials and Methods

### Participants and Procedure

In our center, all donors and recipients have consultations with a nephrologist, a nurse practitioner, a transplant coordinator, and a social worker, in which written and verbal information about the donation/transplantation process and accompanying risks and consequences is provided. The written information is provided in the native language of the donor/recipient when possible. Subsequently, the prospective donors and recipients sign an informed consent form for the donation/transplantation procedure.

Between 19 April 2011 and 28 February 2012, all prospective living kidney donors and living donor kidney recipients who were hospitalized for living donation or transplantation at Erasmus Medical Center in Rotterdam were invited to participate in this study on the day of admission into the hospital (1 day before donation/transplantation). The participants were informed about the study and were asked to complete a written questionnaire in their native language (see below). Under Dutch law, simple questionnaire-based investigations do not need approval of a medical ethical committee.

In addition, we used data from Ismail et al. [26] consisting of R3K-T scores of a representative sample from the general Dutch population (*n* = 515) that completed the questionnaire in an online survey. For more details about this sample see Ismail et al. [26].

### Measurements

#### Sociodemographic Characteristics

The following sociodemographic characteristics were obtained from medical records: age, gender, marital status, employment status, highest level of education completed, native country, native language, religious affiliation, and registration in the Dutch organ donation register. Finally, we assessed co-habitation by asking if the donor and recipient lived in the same house (yes/no). Co-habitation was included as indicator for the closeness of the relationship between donor and recipient that could influence the knowledge level of the participants. See Table 1 for details.

**Table 1:** Participants’ sociodemographic characteristics and medical factors

	Recipients			Donors		
	Participants (*n* = 81)	Non-participants (*n* = 31)	*p* value	Participants (*n* = 85)	Non-participants (*n* = 30)	*p* value
	*n* (%)	*n* (%)		*n* (%)	*n* (%)	
Median age (range)	55 (19–77)	55 (22–79)	0.894	49 (21–86)	53 (21–83)	0.588
Gender			0.640			0.985
Men	56 (69.1)	20 (64.5)		37 (43.5)	13 (43.3)	
Marital status			0.075			0.562
Married/living together	58 (71.6)	17 (54.8)		60 (70.6)	20 (66.7)	
Single/divorced/widowed	22 (27.2)	14 (45.2)		23 (27.1)	10 (33.3)	
Missing	1 (1.2)	0		2 (2.4)	0	
Employment status			0.439			0.366
Paid employment	37 (45.7)	11 (35.5)		50 (58.8)	15 (50.0)	
Retired/voluntary work/unemployed	43 (53.1)	18 (58.1)		34 (40.0)	15 (50.0)	
Missing	1 (1.2)	2 (6.5)		1 (1.2)	0	
Highest level of education completed			0.786			0.964
Primary/secondary school	33 (40.7)	14 (45.2)		36 (42.4)	13 (43.3)	
Further education	45 (55.6)	17 (17.6)		48 (56.5)	17 (56.7)	
Missing	3 (3.7)	0		1 (1.2)	0	
Native country			0.641			0.119
The Netherlands	62 (76.5)	25 (80.6)		73 (85.9)	22 (73.3)	
Other country	19 (23.5)	6 (19.4)		12 (14.1)	8 (26.7)	
Native language			0.558			0.131
Dutch	69 (85.2)	25 (80.6)		74 (87.1)	23 (76.7)	
Non-Dutch	12 (14.8)	6 (19.4)		10 (11.8)	7 (23.3)	
Missing				1 (1.2)	0	
Religious affiliation			0.265			0.837
Yes	50 (61.7)	15 (48.4)		53 (62.4)	19 (63.3)	
No	31 (38.3)	15 (48.4)		28 (32.9)	11 (36.7)	
Missing	0	1 (3.2)		4 (4.7)	0	
Registered in Dutch organ donation register (deceased donation)			0.072			0.230
Yes	26 (32.1)	11 (35.5)		32 (37.6)	6 (20.0)	
No	50 (61.7)	15 (48.4)		50 (58.8)	22 (73.3)	
Missing	5 (6.2)	5 (16.1)		3 (3.5)	2 (6.7)	
Co-habitation			0.222			0.677
Yes	29 (35.8)	15 (48.4)		33 (38.8)	10 (33.3)	
No	52 (64.2)	16 (51.6)		52 (61.2)	19 (63.3)	
Missing				0	1 (3.3)	
Cause of kidney failure			0.967			
Inherited disease	20 (24.7)	9 (29.0)				
Non-inherited disease	37 (45.7)	17 (54.8)				
Missing	24 (29.6)	5 (16.1)				
Pre-emptive transplantation			0.117			
Yes	31 (38.3)	7 (22.6)				
Number of transplants			0.475			
First transplantation	69 (85.2)	28 (90.3)				
Re-transplantation (>1)	12 (14.8)	3 (9.7)				

#### Knowledge Level

The Rotterdam Renal Replacement Knowledge-Test (R3K-T: [26–28]) was used to measure knowledge about dialysis, transplantation, and living donation. The items of the questionnaire were based on literature as well as on contributions of experts and patients who were involved during the item generation process [26]. The questionnaire consists of 21 items and takes 10–15 min to complete. The scale comprises two subscales: the first subscale is *dialysis and transplantation* (*DT*) and consists of 11 items, and the second subscale is *living donation* (*LD*) and consists of 10 items. The test is available in nine languages: Dutch, English, French, Spanish, Arabic, Turkish, Papiamento, Portuguese, and Modern Hindi, which are the most commonly spoken languages in the Rotterdam region. In cases of doubt about an answer, the participants were asked to answer with “I do not know”; this was scored as an incorrect answer. Correct answers were assigned a score of one and summed per subscale. The scores on the subscales were summed to calculate a total score.

The R3K-T has been validated in 187 patients on dialysis, 82 patients who were undergoing live donor kidney transplantation the following day, and the Dutch (*n* = 515) and American general population (*n* = 550) [26]. In the present study, we explored the knowledge of the same population of 82 patients who were undergoing live donor kidney transplantation the following day with their associated living donors. We excluded one recipient of this population for our analysis, because this person did not meet our criterion of completion of 70 % of the questionnaire.

#### Medical Factors

Medical factors that are indicators of experience with ESRD and RRT were obtained from the recipients’ medical records: whether the cause of kidney failure was an inherited disease (yes/no), whether the patient was on dialysis prior to transplantation (yes/no), and whether this transplantation was the first transplantation or a re-transplantation. Transplantation without previous dialysis is called “pre-emptive transplantation”. See Table 1 for details.

### Statistical Analyses

Firstly, we examined whether sociodemographic characteristics differed between participants versus non-participants (donors and recipients who refused to participate or were not approached due to logistical issues) using independent *t* tests for continuous data and chi-square tests for categorical data.

Secondly, to examine whether there were gaps in knowledge among prospective living kidney donors and recipients, their scores were compared with knowledge scores of the general Dutch (naïve) population. Boxplots were made for the three groups on the two subscales. Then, we examined how many donors and recipients had scores that resemble the knowledge of the naïve population better than the knowledge of their own population. We classified the scores of donors and recipients using cutoff points as calculated by the c-formula of Jacobson and Truax [29]. We calculated the cutoff point for the donor population using the means and standard deviations of the donor population and the naïve population on the R3K-T, resulting in a cutoff point *c* = 12. If a donor has a R3K-T score lower than 12, his/her knowledge level is more comparable with the knowledge level of the naïve population rather than the donor population. Cutoff points for the recipient population reported by Ismail et al. [26] were used for the recipients: the cutoff point between the naïve population and dialysis patients is *c* = 11, the cutoff point between dialysis patients and the recipient population is *c* = 14.

Thirdly, to investigate which items were not well understood by the donors and recipients, we calculated the percentages of the donors and recipients who answered the question incorrectly or did not know the answer.

Finally, we conducted a multivariate analysis of variance (MANOVA) to examine whether recipients and donors had different knowledge levels on the two subscales, and to examine whether sociodemographic variables and medical factors were associated with the scores on the two subscales. Before conducting the MANOVA, three steps were taken. In the first step, we screened the donors’ and recipients’ knowledge scores for outliers on the total score: z-score >3.29 [30]. In the second step, the scores were reversed and subsequently transformed using a square root transformation, as the donors’ and recipients’ knowledge scores were not normally distributed. We note that all data reported in this article is transformed back and can be interpreted as the original scores. The final step were univariate analyses (Pearson’s correlations for the continuous variables and independent *t* tests for the categorical variables) to select the covariates that had a potential relationship with knowledge on DT and LD (*p* < 0.10). In a primary analysis, a MANOVA was conducted with knowledge on DT and LD as dependent variables, and the group factor (donors vs. recipient) as well as the selected sociodemographic variables as independent variables. In a secondary analysis among recipients only, the selected medical factors were added as covariates. Significant covariates in the MANOVA were followed up using univariate ANOVA’s.

## Results

### Participants

Between 19 April 2011 and 28 February 2012, 115 living kidney donors and 115 living donor kidney recipients were hospitalized for living donation or transplantation. Two recipients were children and were excluded from this study. One recipient was re-transplanted within the research period and was only approached for participation at the first transplantation. Due to logistical issues such as last minute changes in theater planning, we were unable to collect data from 25 recipients and 22 donors. Seven donors refused participation: three donors gave no reason and four donors reported that they were too strained because of their hospitalization. This last reason was also mentioned by five recipients who refused participation. One recipient and one donor completed less than 70 % of the questionnaire (15 items or less) and were excluded from the analyses. Consequently, 81 living donor kidney recipients and 85 living kidney donors were studied.

### Sociodemographic and Medical Characteristics

Table 1 shows the sociodemographic characteristics and medical factors of the 81 prospective living donor kidney recipients and the 85 living kidney donors who participated in this study. Most recipients were male, and over half of the donors were female. Over half of the recipients and donors were married or living together, were well educated, had been born in the Netherlands, had Dutch as their native language, had a religious affiliation, and were not registered in the Dutch organ donation register. Over half of the donors and recipients did not live in the same house. Non-inherited diseases were the most common cause of kidney failure. Most recipients were on dialysis before their transplantation and were about to undergo primary transplantation.

Table 1 also shows the sociodemographic and medical characteristics of the non-participants and a comparison of these characteristics with the participants’ characteristics. Recipients and donors who participated in this study did not differ from recipients and donors who did not participate on all sociodemographic characteristics and medical factors. We concluded that our study population was a representative sample of the donor and recipient population at Erasmus Medical Center.

The participants completed the questionnaire in their own language: one donor completed the questionnaire in English and one recipient in Turkish, the remaining participants completed the questionnaire in Dutch.

### Gaps in Knowledge

**Fig.1:**
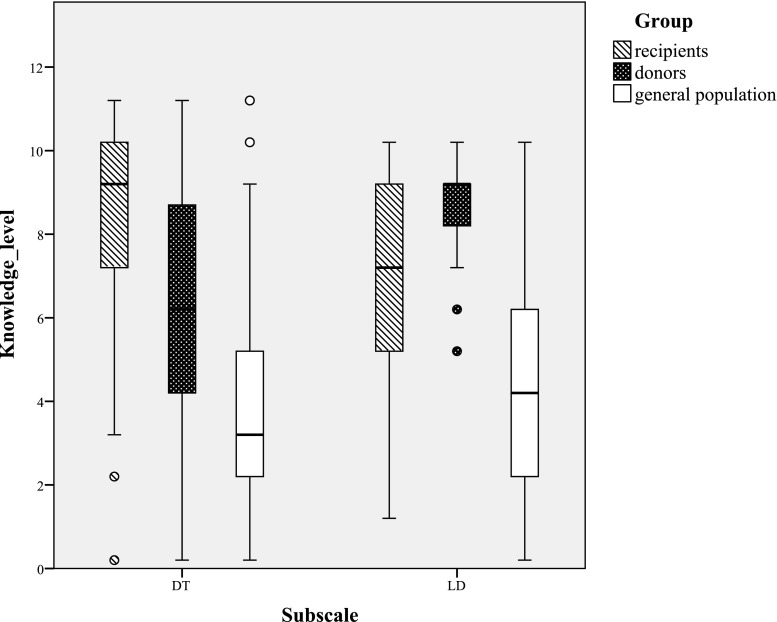
The boxplots for the knowledge scores of the recipients, donors, and the general population

The boxplots for the knowledge scores of the recipients, donors, and the general population (Fig. 1) confirm our finding that donors and recipients differed on their knowledge level. In addition, the boxplots make clear that within the three groups, participants varied widely in their knowledge, except for donors’ scores on LD.

Thirteen of the 85 donors (15.3 %) had a total score lower than 12, the cutoff point between the naïve population and the donor population. That means that 15.3 % of the donors had a score that is comparable with the knowledge level of the naïve population. Fourteen of the 81 recipients (17.3 %) had a total score lower than 11, the cutoff point between the naïve population and the dialysis patient population. That means that 17.3 % of the recipients had a score that is comparable with the knowledge level of the naïve population rather than that of the patient populations. Nine of the 81 recipients (11.1 %) had a total score between 11 (the cutoff point between the naïve population and the dialysis patient population) and 14 (the cutoff point between the dialysis patient population and the recipient population). That means that 11.1 % of the recipients had a score that resembles the knowledge of the dialysis patient population better than the knowledge of the recipient or the naïve population.

Tables 2 and 3 show the percentages of donors and recipients who answered the items incorrectly or did not know the answer. The percentages reveal that donors scored the lowest on items relating to peritoneal dialysis. Recipients scored the lowest on items relating to the health consequences of donation for donors. A substantial minority of donors and recipients answered items concerning the consequences of living kidney donation incorrectly or did not know the answer (item 12–17).

**Table 2:** Percentages of the donors/recipients who answered items incorrectly or did not know the answer on the subscale “dialysis and transplantation”

Subscale DT	Item	Correct answer	Recipients	Donors
1	Peritoneal dialysis is a form of dialysis for treating patients with end-stage renal disease. Which part of the body makes this treatment possible?	The peritoneum	18.5	52.9
2	Peritoneal dialysis is a form of renal replacement therapy that can be used as an alternative for hemodialysis. An advantage of peritoneal dialysis is:	That you have more freedom of movement in between the in and out flow of the dialysis fluid.	38.3	64.7
3	During peritoneal dialysis, fluid is brought into the abdominal cavity through a catheter. What happens with the fluid after that?	The fluid stays in the abdominal cavity, after a couple of hours it is removed.	44.4	64.7
4	Peritonitis is an infection of the peritoneum. This is one of the biggest problems with patients with peritoneal disease.	True	42.0	58.8
5	Certain vitamins are lost during dialysis. If you are on dialysis you are therefore prescribed extra vitamins.	True	23.5	60.0
6	How many hours a day is a hemodialysis patient connected to the machine?	3–8	12.3	29.4
7	Renal replacement therapy is necessary if kidney function is only 50 %.	False	25.9	34.1
8	To be connected to the hemodialysis machine, there must be permanent access to the bloodstream.	True	13.6	32.9
9	Kidneys from living donors have a longer graft survival rate than kidneys from deceased donors.	True	17.3	23.5
10	Kidney transplantation is generally preferred to dialysis for the treatment of end-stage renal disease.	True	11.1	18.8
11	Immunosuppressive drugs are administered to transplant patients for:	Prevention and treatment of rejection of the kidney.	13.6	23.5

**Table 3:** Percentages of the donors/recipients who answered items incorrectly or did not know the answer on the subscale “living donation”

Subscale LD	Item	Correct answer	Recipients	Donors
12	Surgical complications after donation are common in living kidney donors.	False	35.8	11.8
13	Donating a kidney increases the risk of developing a kidney disease.	False	18.5	7.1
14	Most living kidney donors remain in the hospital for 2 weeks after surgery.	False	32.1	4.7
15	Very few living kidney donors have long-term health problems after donation.	True	46.9	31.8
16	Kidney donation may affect a woman’s chance of getting pregnant.	False	59.3	47.1
17	Most living kidney donors can participate in sports and work within 4–6 weeks after donation.	True	22.2	14.1
18	When the kidney of a living donor does not match the recipient, living donation is no longer an option with this donor.	False	38.3	27.1
19	A living kidney donor has to be younger than 50 years old.	False	21.0	9.4
20	Only direct family members (brothers, sisters, parents, or children) can donate a living kidney.	False	13.6	1.2
21	All the hospital costs of a living kidney donation are paid for by the recipient’s health insurance and not by the donor’s insurance.	True	19.8	9.4

### MANOVA’s

#### Primary Analysis

The donors’ and recipients’ scores were screened for outliers on the total score, and one donor was deleted. The following sociodemographic variables had a potential relationship with knowledge on DT and LD and were entered into the MANOVA (*p* < 0.10): group, marital status, employment status, religious affiliation, and native language. Using the Pillai’s trace, there was a significant group effect on knowledge, *V* = 0.30, *F*(2,151) = 31.85, *p* < 0.001. There was also a significant association between native language and knowledge on DT and LD, *V* = 0.04, *F*(2,151) = 3.14, *p* < 0.05. Marital status, employment status, and religious affiliation were not related to knowledge on DT and LD. Univariate ANOVA’s showed that donors and recipients differed on both subscales (see Table 4): recipients knew more about DT than donors (*F*(1,152) = 24.03, *p* < 0.001) and donors more about LD than recipients (*F*(1,152) = 19.32, *p* < 0.001). Univariate ANOVA’s also showed that participants whose native language was Dutch knew more about DT than participants whose native language was not Dutch (*F*(1,152) = 4.01, *p* < 0.05). The same effect was found on knowledge about LD (*F*(1,152) = 4.36, *p* < 0.05).

**Table 4:** Univariate ANOVA’s of significant covariates in the primary MANOVA

	Subscale 1: dialysis and transplantation				Subscale 2: living donation			
	Estimates				Estimates			
	CI				CI			
	Lower bound	Upper bound	*F*	*p*	Lower bound	Upper bound	*F*	*p*
Group			24.03	<0.001			19.32	<0.001
Recipients	6.62	8.06			5.38	6.54		
Donors	4.21	6.13			6.72	7.74		
Native language			4.01	0.047			4.36	0.038
Dutch	6.53	7.52			6.78	7.43		
Non-Dutch	4.12	6.94			5.17	7.01		

A sensitivity analysis with the outlier included in the MANOVA revealed the same results. The MANOVA was also repeated with interactions between “group” and the remaining covariates as independent variables to examine whether the effects differed across donors and recipients; however, none of these covariates were significant.

#### Secondary Analysis

Only the medical factor “pre-emptive transplantation” had a potential relationship with knowledge and was added into the MANOVA (*p* < 0.10). In the MANOVA, pre-emptive transplantation was also associated with knowledge, *V* = 0.25, *F*(2,72) = 11.95, *p* < 0.001. Univariate ANOVA’s showed that this relationship was only significant for knowledge about DT (*F*(1,73) = 12.37, *p* < 0.01) and not for LD, *F*(1,73) = 1.48, *p* = 0.23 (See Table 5). However, as the univariate ANOVA of DT violated the homogeneity of variance assumption, the test was repeated with a Welch’s test and showed the same results, *F*(1,53.61) = 6.69, *p* < 0.05. The relationship found included that recipients knew less about DT is they were undergoing pre-emptive transplantation. We note that the relationship between knowledge and native language was not significant in this MANOVA, *V* = 0.05, *F*(2,72) = 1.76, *p* = 0.18.

**Table 5:** Univariate ANOVA’s of significant covariates in the secondary MANOVA

	Subscale 1: dialysis and transplantation				Subscale 2: living donation			
	Estimates				Estimates			
	CI				CI			
	Lower bound	Upper bound	*F*	*p*	Lower bound	Upper bound	*F*	*p*
Pre-emptive transplantation			12.37	0.001			1.48	0.227
Yes	4.93	7.34			5.35	7.35		
No	7.30	8.84			4.82	6.62		

## Discussion

The results of the present study give insight into how informed prospective living kidney donors and living donor kidney recipients are when they completed the informed consent procedure. We found that a number of donors and recipients did not retain all the information they were given as they had incomplete knowledge about dialysis, transplantation, and living donation at the time of their surgery. Furthermore, donors and recipients retained different information: recipients knew significantly more about dialysis and transplantation than donors, and donors knew significantly more about living donation than recipients. Finally, recipients and donors knew less about DT and LD if their native language was not Dutch and recipients knew less about DT if they were undergoing pre-emptive transplantation.

It appears that, even though prospective living kidney donors and recipients are informed and go through the donation process together, they retain different information. This might stem from selective attention for personally relevant information during information gathering. Psychological research has shown that selective attention for personally relevant information strengthens the encoding and retrieval of this information [31].

In addition, we found that 15 % of the donors and 17 % of the recipients had a score that resembles the knowledge of the naïve general population better than their own population, i.e., they had a significantly lower knowledge level than one would expect. Moreover, we found that a substantial minority of donors and recipients lacked knowledge about the risks of living donation (items 12–17). Results consistent with these findings were found among living liver donors [32]. Valapour and colleagues found in a retrospective study that living kidney donors reported after donation that they lacked knowledge about the risks of donation if they were asked how informed they were at the time of their surgery [20]; however, subsequent experiences could have biased their answers. We found consistent results using an objective measure and at a moment in time when donors are still unbiased by the experience, and subsequently, we add that this is also the case for prospective recipients. Valapour and colleagues [20] concluded that the motivation of potential living kidney donors for donating their kidney is based mostly on a “wish to help” [18] rather than on their understanding of the risks and benefits of donation [20, 33, 34]. A study by Papachristou and colleagues [19] revealed consistent motivations for donation among prospective living liver donors, and also found that a proportion of donors avoided later reconsideration or confrontation with donation-related issues. These studies indicate that the motivations of prospective living kidney donors may lie more in emotional considerations than in rational ones which may influence information retention. It is also possible that some recipients avoid confrontation with donation-related issues. We speculate that this could be the consequence of an avoidant coping style of prospective recipients who have some difficulties in accepting a kidney from their potential donor [35].

These findings raise the question of the potential consequences of a lack of knowledge about the risks of donation/transplantation. Accurate knowledge about a prospective event contributes to realistic expectations and may prevent potential disappointment [36]. Johnson et al. [9] found some indication for this relationship among living kidney donors by showing that donors who experienced the least amount of stress reported that they were well informed and knew what to expect before and after donation. Whether incomplete objective knowledge about the consequences of the donation/transplantation contributes to disappointment and/or stress after donation/transplantation among donors and recipients, needs further research.

Our results show that donors’ and recipients’ knowledge levels were particularly lower if their native language was not Dutch and therefore deviated from the professional’s native language. This result is probably not the consequence of linguistic barriers, as most participants in our study speak the Dutch language fluently: only one donor and one recipient completed the R3K-T in another language than Dutch. It is possible that cultural factors played a role. A review by Schouten et al. [37] showed that if doctors and patients have different cultural and ethnic backgrounds, doctors interact less affectively with the patient and the patients are less assertive and affective during the medical consultations than in case of equal backgrounds. This phenomenon could be the consequence of factors like differences in beliefs about illness and values across cultures, e.g., the perceived appropriateness to talk about illness and ask questions. It is possible that difficulties in the doctor-patient communication contributed to lower knowledge among ethnic minorities in our study; however, this requires further research. As earlier studies also revealed that patients from ethnic minorities are less likely to pursue with live donor kidney transplantation [38], our findings highlight that even if these patients proceed with live donor kidney transplantation, extra care must be taken to ensure full comprehension of information that forms the basis of informed consent. We recommend that if the donor’s or recipient’s native language deviates from the professional’s native language regardless of speaking a common language, extra efforts should be made to ensure that they understand the information they are given and professionals should be sensitive for the potential influence of cultural differences during medical consultations.

A striking result of this study is that native language was associated with knowledge in the primary analysis with donors and recipients included, but not in the secondary analysis with only recipients and the covariate “pre-emptive transplantation” included. As we found no difference between donors and recipients in the association between native language and knowledge, we conclude that “pre-emptive transplantation” is probably more strongly associated with knowledge about DT than native language.

Although this study has several strengths, a number of limitations have to be noted. Firstly, knowledge was measured on the day of admission into the hospital, which is a stressful day for some donors and recipients. Possibly a number of participants experienced stress at the time of completing the questionnaire that influenced the recall of knowledge; however, this requires further research. Secondly, as the education process of potential living kidney donors may differ across transplant centers and countries, our findings may not be generalizable to other settings and requires similar research in other countries.

A practical contribution of the present study is the norm scores of actual donors on the R3K-T. The boxplots and cutoff points can be used to determine how a donor scored relative to other donors, recipients, or a naïve population. These insights can be used to determine whether a potential donor or recipient needs extra education. The questionnaire could also be used to examine whether knowledge of potential donors who decided not to donate differ from knowledge of actual donors. The results of such studies could clarify whether a lack of knowledge is a barrier to pursuing living kidney donation, which has been found among potential recipients of live donor kidney transplantation [23].

In conclusion, potential living kidney donors and living donor kidney recipients retain different information during the information process of living donation/transplantation. The decision to undergo living donation/transplantation appears to be not always based on full knowledge of the risks. We recommend that professionals assess knowledge and information needs of prospective donors and recipients using the R3K-T in order to tailor educational efforts to the informational needs of these individuals, and extra attention is required for non-native speakers.

## References

[1] Spital A (2005). Increasing the pool of transplantable kidneys through unrelated living donors and living donor paired exchanges. Semin Dial.

[2] Vollmer WM, Wahl PW, Blagg CR (1983). Survival with dialysis and transplantation in patients with end-stage renal disease. N Engl J Med.

[3] Segev DL, Muzaale AD, Caffo BS, Mehta SH, Singer AL, Taranto SE (2010). Perioperative mortality and long-term survival following live kidney donation. JAMA.

[4] Matas AJ, Bartlett ST, Leichtman AB, Delmonico FL (2003). Morbidity and mortality after living kidney donation, 1999–2001: survey of United States transplant centers. Am J Transplant.

[5] Johnson EM, Remucal MJ, Gillingham KJ, Dahms RA, Najarian JS, Matas AJ (1997). Complications and risks of living donor nephrectomy. Transplantation.

[6] Mjøen G, Hallan S, Hartmann A, Foss A, Midtvedt K, Oyen O (2014). Long-term risks for kidney donors. Kidney Int.

[7] Muzaale AD, Massie AB, Wang M, Montgomery RA, McBride MA, Wainright JL (2014). Risk of end-stage renal disease following live kidney donation. JAMA.

[8] Ibrahim HN, Foley R, Tan L, Rogers T, Bailey RF, Guo H (2009). Long-term consequences of kidney donation. N Engl J Med.

[9] Johnson EM, Anderson JK, Jacobs C, Suh G, Humar A, Suhr BD (1999). Long-term follow-up of living kidney donors: quality of life after donation. Transplantation.

[10] Fournier C, Pallet N, Cherqaoui Z, Pucheu S, Kreis H, Mejean A (2012). Very long-term follow-up of living kidney donors. Transpl Int.

[11] Dols LF, Ijzermans JN, Wentink N, Tran TC, Zuidema WC, Dooper IM (2010). Long-term follow-up of a randomized trial comparing laparoscopic and mini-incision open live donor nephrectomy. Am J Transplant.

[12] Barri Y, Parker Iii T, Kaplan B, Glassock R (2009). Primum non nocere: is chronic kidney disease staging appropriate in living kidney transplant donors?. Am J Transplant.

[13] Henderson AJZ, Landolt MA, McDonald MF, Barrable WM, Soos JG, Gourlay W (2003). The living anonymous kidney donor: lunatic or saint?. Am J Transplant.

[14] Jowsey SG, Schneekloth TD (2008). Psychosocial factors in living organ donation: clinical and ethical challenges. Transplant Rev (Orlando).

[15] Marteau TM, Dormandy E, Michie S (2001). A measure of informed choice. Health Expect.

[16] Abecassis M, Adams M, Adams P (2000). Consensus statement on the live organ donor. JAMA.

[17] Lopp L (2013). Regulations regarding living organ donation in Europe. Possibilities in harmonisation.

[18] Lennerling A, Forsberg A, Meyer K, Nyberg G (2004). Motives for becoming a living kidney donor. Nephrol Dial Transplant.

[19] Papachristou C, Walter M, Frommer J, Klapp BF (2010). Decision-making and risk-assessment in living liver donation: how informed is the informed consent of donors? A qualitative study. Psychosomatics.

[20] Valapour M, Kahn JP, Bailey RF, Matas AJ (2011). Assessing elements of informed consent among living donors. Clin Transplant.

[21] Vamos EP, Novak M, Mucsi I (2009). Non-medical factors influencing access to renal transplantation. Int Urol Nephrol.

[22] Martin P. Living donor kidney transplantation: preferences and concerns amongst patients waiting for transplantation in New Zealand. J Health Serv Res Policy 2013; in press. 10.1177/1355819613514957 24366157

[23] Rodrigue JR, Cornell DL, Lin JK, Kaplan B, Howard RJ (2007). Increasing live donor kidney transplantation: a randomized controlled trial of a home-based educational intervention. Am J Transplant.

[24] Vamos EP, Csepanyi G, Zambo M (2009). Sociodemographic factors and patient perceptions are associated with attitudes to kidney transplantation among haemodialysis patients. Nephrol Dial Transplant.

[25] Waterman AD, Peipert JD, Hyland SS, McCabe MS, Schenk EA, Liu J (2013). Modifiable patient characteristics and racial disparities in evaluation completion and living donor transplant. Clin J Am Soc Nephrol.

[26] Ismail SY, Timmerman L, Timman R (2013). A psychometric analysis of the Rotterdam Renal Replacement Knowledge-Test (R3K-T) using item response theory. Transpl Int.

[27] Ismail SY, Massey EK, Luchtenburg AE, et al. Development of the Rotterdam Renal Knowledge-Test (R3K-T). 2011. http://repub.eur.nl/pub/23968/Manuscript_Development%20of_R3K-T.pdf. Accessed 31 March 2014.

[28] Dobbels F, Duerinckx N (2013). Wise decisions on renal replacement therapy require knowledgeable patients and good self-report knowledge scales. Transpl Int.

[29] Jacobson NS, Truax P (1991). Clinical significance: a statistical approach to defining meaningful change in psychotherapy research. J Consult Clin Psychol.

[30] Field A (2009). Discovering statistics using SPSS.

[31] Gazzaley A, Nobre AC (2012). Top-down modulation: bridging selective attention and working memory. Trends Cogn Sci.

[32] Gordon EJ, Daud A, Caicedo JC (2011). Informed consent and decision-making about adult-to-adult living donor liver transplantation: a systematic review of empirical research. Transplantation.

[33] Gordon EJ (2012). Informed consent for living donation: a review of key empirical studies, ethical challenges and future research. Am J Transplant.

[34] Stothers L, Gourlay WA, Liu L (2005). Attitudes and predictive factors for live kidney donation: a comparison of live kidney donors versus nondonors. Kidney Int.

[35] Pradel FG, Limcangco MR, Mullins CD, Bartlett ST (2003). Patients’ attitudes about living donor transplantation and living donor nephrectomy. Am J Kidney Dis.

[36] Ursin H, Eriksen HR (2004). The cognitive activation theory of stress. Psychoneuroendocrinology.

[37] Schouten BC, Meeuwesen L (2006). Cultural differences in medical communication: a review of the literature. Patient Educ Couns.

[38] Roodnat JI, van de Wetering J, Zuidema W, van Noord MA, Kal-van Gestel JA, IJzermans JN (2010). Ethnically diverse populations and their participation in living kidney donation programs. Transplantation.

